# Supplementary material for: Fast semantic segmentation method for machine vision inspection based on a fewer-parameters atrous convolution neural network
Source: PLoS One. 2021 Feb 10;16(2):e0246093. doi: 10.1371/journal.pone.0246093 (PMC7875430; doi:10.1371/journal.pone.0246093)
Supplement: Attachment — Submitted filename: 0903Reply to Review Reports.pdf [file pone.0246093.s001.pdf]

Reply to Review Reports

Dear Editor,

This article have sent to Plos One as Semantic Segmentation Visual Detection Technology Based on Downsampling Porous Convolution Architecture Network Model Lightweight Optimization (ONE-D-20-15502), and receive a decision letter.

Thank you very much for your time and discussion on this manuscript. We also thank the referees very much for their constructive comments. We have revised the manuscript accordingly. The replies are follows:

Additional Editor Comments (if provided):

1. *Seeing the comments of the reviewers, it seemed that rigorous statistical is required.*

Reply: According to the editor comment, we follow reviewer#1 's suggestion **3** and reviewer#2 's suggestion **2**, *to make sure statistical analysis is performed appropriately and rigorously.*

2. *Also, detailed comparison with DeepLab v3 should be added to improve the quality of the work to convince the results.*

Reply: According to the editor comment, we follow reviewer#2 's suggestion **1**, add comparison with DeepLab v3, to improve the quality of the work to convince the results.

Reviewer #1:

This work proposes various tunings to reduce the time-consumption of existing studies in the semantic segmentation task, and conducted various experiments to find the structure that gives the best performance through the atrous convolution architecture and parameter tuning of residual building blocks.

Here are some of my major comments (but are not limited).

1. *In page 6, The explanation of the formula described in the second and third paragraphs of the proposed method is insufficient.*

In particular, the authors need to describe what PA, d, $T_{seg\text{-}a}$*, and Tseg-t mean in Eq. (1).*

Reply: According to the reviewer's suggestion, we describe what *PA* mean in Eq. (1). We also remove the whole Eq. (2) about *FLOPs*, which consists of ***d***, $T_{seg\text{-}a}$, and $T_{seg\text{-}t}$, because we didn't use *FLOPs* metric in other part of this paper.

2. *In page 7, The authors mentioned that the model is optimized in the direction of minimizing* T_{seg} *while satisfying* IoU_{min}*, but the quantitative value for* IoU_{min} *is not mentioned in the experiment with a specific dataset.*

Reply: According to the reviewer's suggestion, we *mention the quantitative value for* IoU_{min} in the experiment with a specific dataset.

In PASCAL VOC dataset semantic segmentation task, IoU_{min} is equal to 85%, in anti-counterfeit mark semantic segmentation task, IoU_{min} is equal to 90%

3. *In Tables 3 and 4, experiments were performed on the PASCAL VOC and CNY datasets, but not all of the performance metrics for performance comparison were provided. Of course, the results of the two experiments are not directly compared to give a meaningful result, but it is better to provide the same performance metric in all experiments.*

Reply: According to the reviewer's suggestion, we provide all of the performance metrics for performance comparison. And make sure providing the same performance metric in all experiments.

In PASCAL VOC *datasets* semantic segmentation task [1], we add *IoU* on class-level to provide all of the performance metrics for performance comparison.

In CNY datasets semantic segmentation task, we remove the $AP^{IoU=0.1}$ metric, to make sure providing the same performance metric in all experiments.

4.*The detailed explanation of Eq. (3) should be totally revised to convince readers as there are several vague illustrations. For example, in the prapagraph illustrating eq. (3), the intention of using the sentence ".... Backbone network depth ... depend on ...." is very confusing.*

Reply: According to the reviewer's suggestion, we have totally revised The detailed explanation of Eq. (3).

5.*The resolution of the figure is too small. And there are figures that is not mentioned in the text, so it is necessary to describe Figs. 2 to 5.*

Reply: According to the reviewer's suggestion, we describe Figs. 2 to 5. And provide the figure @ 600dpi.

6.*As a minor comment, the English should be improved.*

Reply: According to the reviewer's suggestion, we editing the paper with a professional editors at Editage, to ensure that the language is clear and free of errors. The logical presentation of ideas and the structure of the paper were also checked during the editing process.

Reviewer #2:

This work proposes ensuring the detection accuracy, reduces the detection time with atrous convolution layers

The following is some of my comments but the manuscript should be improved in many other

aspects too.

1. In Tables 3 and 4, the authors used ResNet, ResNet V2 to compare the performance. However, in my opinion, using the original DeepLab V3 is a more preferable for fair and accurate comparison as the authors used DeepLab V3 as their backbone network. Without this, it is very hard to compute the validity and outperformance of their proposal.

Reply: According to the reviewer's suggestion, we using the original DeepLab V3 to compare the performance.

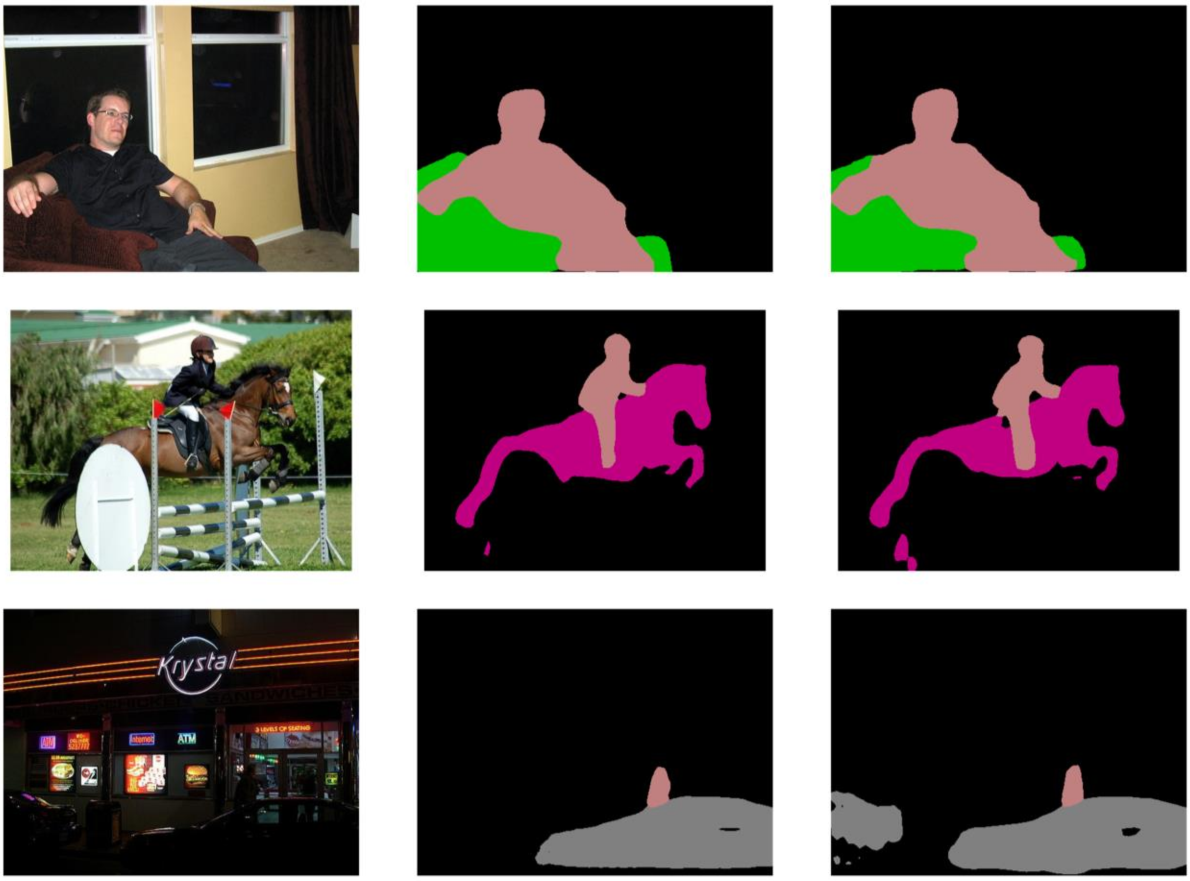

Oringinal image DeepLab v3 with Resnet50 DeepLab v3 with modified Resnet50

Fig 1. PASCAL VOC semantic segmentation by modified DeepLabv3

2. At page 12, the authors were used only CNY datasets for testing, but for more objective comparison to show its outperformance, the comparative results drawn from various segmentation datasets such as COCO, ImageNet, and CIFAR 10 should be inherently required.

Reply: According to the reviewer's suggestion, we pretrain the DeepLab V3 in *COCO, ImageNet, and* add the comparative results drawn from Cityscapes segmentation datasets

TABLE 1. Performances of different methods on the CITYSCAPES pixel-level semantic labeling task.

Method	IoU_{class}(%)	$IoU_{category}$(%)	Segmentation time T_{seg}(ms)
DeepLab [16]	64.5	83.7	——
DeepLab v2 [18]	70.4	86.4	——

DeepLab v3 with ResNet50[19]	78.7	89.8	**494ms**
DeepLab v3 with modified ResNet50[ours]	**79.9**	**91.6**	497ms

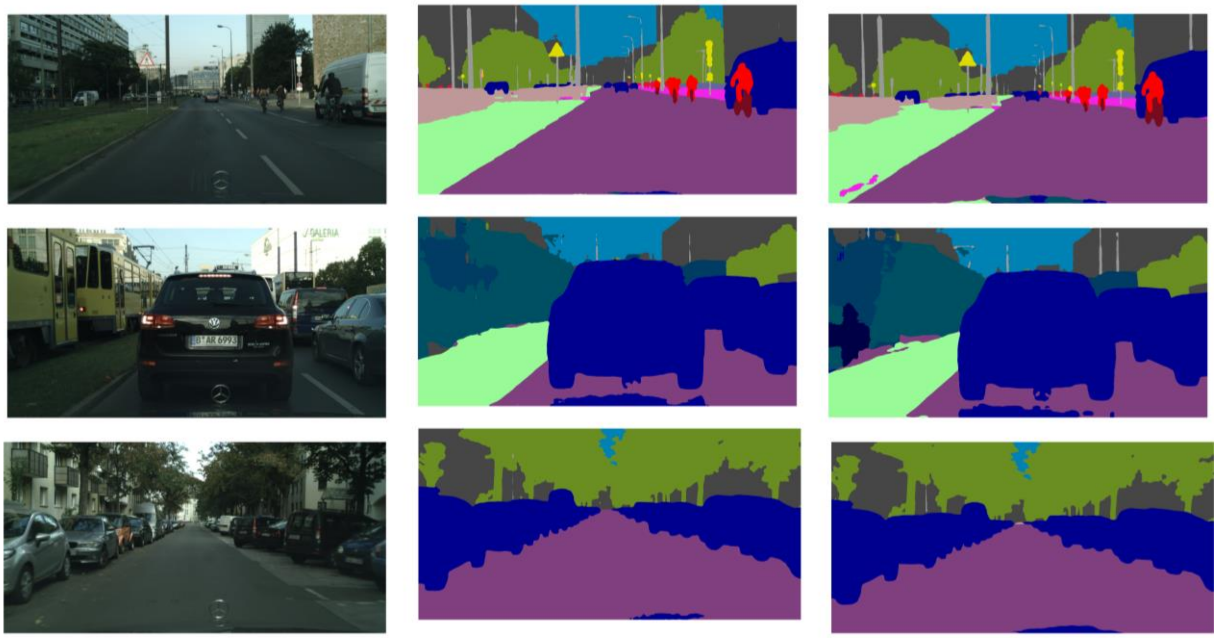

Fig 2. CITYSCAPES pixel-level semantic labeling by modified DeepLabv3

3. The authors need to improve the English grammar for readers to understand better (especially, in their proposed method)

Reply: According to the reviewer's suggestion, we editing the paper with a professional editors at Editage, to ensure that the language is clear and free of errors. The logical presentation of ideas and the structure of the paper were also checked during the editing process.

Others:

We also made all data underlying the findings in our manuscript fully available.

The PASCAL VOC Dataset used to support the findings of this study is available at http://host.robots.ox.ac.uk/pascal/VOC/. The CITYSCAPES Dataset used to support the findings of this study is available at http://cityscapes-dataset.com. The DeepLab v3 pretrained model are the implement of GLUON-CV, and available at https://gluon-cv.mxnet.io/model_zoo/segmentation.html. We provide the DeepLab v3 with modified ResNet50, and a program (*.ipynb) to use it on different image of PASCAL VOC, CITYSCAPES or CNY. https://github.com/HJ0116/utilization_semantic_segmentation
