# Supplementary material for: Fast semantic segmentation method for machine vision inspection based on a fewer-parameters atrous convolution neural network
Source: PLoS One. 2021 Feb 10;16(2):e0246093. doi: 10.1371/journal.pone.0246093 (PMC7875430; doi:10.1371/journal.pone.0246093)
Supplement: Attachment — Submitted filename: Respones to Reviewers.docx [file pone.0246093.s002.docx]

**Respones to Reviewers**

# Dear Editor,

This article have sent to Plos One as Semantic Segmentation Visual Detection Technology Based on Downsampling Porous Convolution Architecture Network Model Lightweight Optimization (ONE-D-20-15502), and receive a decision letter.

Thank you very much for your time and discussion on this manuscript. We also thank the Reviewers very much for their constructive comments. We have revised the manuscript accordingly. The replies are follows:

**Journal Requirements:**

When submitting your revision, we need you to address these additional requirements.

***1.*** *Please ensure that your manuscript meets PLOS ONE's style requirements, including those for file naming.*

**Reply:** According to the editor’s suggestion, we edit our manuscript to meets PLOS ONE's style requirements, including those for file naming.

***2.*** *Please ensure that you refer to Figure 13 in your text as, if accepted, production will need this reference to link the reader to the figure.*

**Reply:** According to the editor’s suggestion, we *refer to Figure 13 in our text***.**

*Additional Editor Comments: Please revise this manuscript to fully respond to reviewer 2's comments.*

**Reply:** According to the editor’s suggestion, we revise this manuscript to fully respond to reviewer 2's comments**.**

**Reviewer #1:**

 In this paper, the authors present fast semantic segmentation method for machine vision inspection based on a fewer parameters atrous convolution neural network. The article is well constructed, the experiments were well conducted, and analysis was well performed.

**Reviewer #2:**

This work designed a fewer-parameters optimization mathematical model according to the atrous convolution architecture network to decrease segmentation time while maintaining the detection accuracy, although there are some significant improvements that the authors reflected previous reviewers, there are several minor aspects the authors should consider:

***1.*** *More recent works should be included, both CNN-based semantic segmentation and Atrous CNN-based works to enlighten your novelty in the related works section.*

**Reply:** According to the reviewer’s suggestion, we list more recent works in the related works section, both CNN-based semantic segmentation and Atrous CNN-based works that enlighten my novelty.

***2.*** *The authors merely presented their observations in the results section. However, to make the experimental results more convinced, please give more explanations on the results you obtained.*

**Reply:** According to the reviewer’s suggestion, we give more explanations on the results that we obtained. Especially the CITYSCAPES pixel-level semantic labeling results and banknote anti-counterfeit segmentation results

***3.*** *Please increase the figures' resolution if possible.*

**Reply:** According to the reviewer’s suggestion, we increase the figures' resolution to 600dpi, which is twice as high as the PLOS ONE requirements.

*While revising your submission, please upload your figure files to the Preflight Analysis and Conversion Engine (PACE) digital diagnostic tool*

**Reply:** As Request, We upload our figure files to the PACE digital diagnostic tool, and upload the PACE output figure files to PLOSONE.
